# Psychiatric staff training in managing medical emergencies: re-audit

**DOI:** 10.1192/bjo.2021.211

**Published:** 2021-06-18

**Authors:** Sarah Abd El Sayed, Sudhir Salujha

**Affiliations:** 1 Stepping Hill Hospital, Laureate House, Wythenshawe Hospital; 2 Stepping Hill Hospital

## Abstract

**Aims:**

In the UK, people with severe mental illness at a greater risk of poor physical health and have higher premature mortality than the general population, highlighting the importance of responding to physical health problems among patients suffering from psychiatric conditions. However, training for staff on inpatient psychiatric units to meet patients’ physical health needs is sometimes overlooked and has not always been effective.

According to NICE Clinical Guideline 25 (2005) and NPSA Rapid Response Report (2008/RRR010), staff on any psychiatric inpatient setting must be capable of monitoring, measurement, and interpretation of vital signs. They must have both adequate information and skills to identify signs indicating worsening of patients’ health and respond effectively to severely ill patients.

Hence, we aim to re-audit the results of a similar audit carried out in 2016 to review the level of medical emergency training (in terms of life support training) of clinical staff across the inpatient psychiatric wards at our local hospital - Stepping Hill Hospital- in Stockport.

Our hypothesis is that there will be a gap in meeting the required standards for training.

**Method:**

A questionnaire including 6 questions (role of the staff member, level of their life support training, when was their training last updated, whether they know the location of the crash trolley, whether they know the local hospital emergency number and whether they should resuscitate the patient if their training is out of date) was given to staff on acute inpatient psychiatric units in Stepping Hill Hospital.

**Result:**

The sample included 49 staff members from all the 3 wards included in the audit. The level of training of nursing staff on the 3 wards was meeting standards except for nursing staff who were new to the wards or coming back to work from prolonged leaves. There was also a gap identified in the level of training of other staff members on the ward as well as on the remaining standards measured by the audit.

**Conclusion:**

A gap was identified in meeting the required standards of training on the inpatient psychiatric units. Reasons identified for this gap are mainly due to the fact that new or bank staff are asked to cover the wards without providing them with appropriate training and without orientating them about the location of different equipments and policies on the ward.

